# Evaluating lactation performance of multiparous dairy cattle to prepartum and/or postpartum supplementation of fat-embedded calcium gluconate

**DOI:** 10.1093/tas/txad104

**Published:** 2023-08-31

**Authors:** D J Seymour, M V Sanz-Fernandez, J B Daniel, J Martín-Tereso, J Doelman

**Affiliations:** Ruminant Research Centre, Trouw Nutrition R&D, PO Box 299, 3800 AG, Amersfoort, The Netherlands; Ruminant Research Centre, Trouw Nutrition R&D, PO Box 299, 3800 AG, Amersfoort, The Netherlands; Ruminant Research Centre, Trouw Nutrition R&D, PO Box 299, 3800 AG, Amersfoort, The Netherlands; Ruminant Research Centre, Trouw Nutrition R&D, PO Box 299, 3800 AG, Amersfoort, The Netherlands; Ruminant Research Centre, Trouw Nutrition R&D, PO Box 299, 3800 AG, Amersfoort, The Netherlands

**Keywords:** energy, hindgut, prebiotic

## Abstract

Prebiotic compounds may be supplemented in the diet to improve animal health and performance in a variety of ways. In dairy cattle, the transition from pregnancy through parturition and lactation represents a critical life stage with many concurrent stressors. The objectives of this study were to evaluate responses to the provision of a hindgut-targeted prebiotic compound (calcium gluconate; HFCG) when supplemented prepartum and/or postpartum in a 2 × 2 factorial design. One hundred and sixty-four multiparous Holstein cattle were enrolled and followed from approximately 21 d prior to calving until 100 d of lactation. Treatments were administered as a pelleted compound feed offered in the rotary milking parlor once daily prepartum and thrice daily postpartum. Information pertaining to milk production and body weight were automatically recorded by the milking equipment, and information pertaining to reproductive and health performance was recorded by farm staff. Cattle that received HFCG prepartum were confirmed pregnant approximately 21 d earlier (*P* = 0.024). Cattle that received HFCG both pre- and postpartum had 9% to 10% higher yields of milk protein, fat, and energy-corrected milk (*P* ≤ 0.037) from weeks 4 to 9 of lactation relative to those that received HFCG exclusively prepartum. Conversely, cattle that received HFCG exclusively postpartum had 9% to 10% higher yields of milk protein, fat, and energy-corrected milk (*P* ≤ 0.037) from weeks 9 to 14 of lactation relative to those that received exclusively the negative control in both periods. The mechanism underlying these responses remains unclear, however, we hypothesize that these responses are due to localized reductions in inflammation in the gut and/or signaling to extragastrointestinal tissues altering energy partitioning and balance.

## Introduction

During the transition from pregnancy through calving and into lactation, the concurrent onset of multiple stressors (e.g., uterine involution, social stress, and reduced voluntary feed intake) can disrupt gastrointestinal barrier function, increasing the permeability to xenobiotic compounds, and potentially contributing to systemic inflammation (Bradford et al., 2015; Sanz-Fernandez et al., 2020). While the rumen is the main site of digestive fermentation in ruminants, a significant amount of energy can still be obtained from fermentation in the hindgut (~8% to 9% ME intake; Siciliano-Jones and Murphy, 1989; Bergman, 1990) and much like the rumen, may also be susceptible to adverse health events (Petri et al., 2021; van Gastelen et al., 2021; Abeyta et al., 2022).

Prebiotic compounds specifically targeting the postruminal digestive tract could prove beneficial during the transition period by supporting animal health and performance (Gibson et al., 2004; Gaggia et al., 2010; Uyeno et al., 2015). Our research group has previously investigated lactating dairy cattle the application of gluconic acid, a prebiotic compound that is fermented to acetate and/or butyrate in the hindgut (Asano et al., 1994; Tsukahara et al., 2002, 2006). We have observed an increase in milk fat yield when post-ruminally infusing calcium gluconate, a salt of gluconic acid (Doelman et al., 2019; McKnight et al., 2019), while no differences were observed when calcium gluconate was mixed directly into the diet (McKnight et al., 2019). In an effort to prevent ruminal degradation of the product, we developed a form of calcium gluconate embedded in a matrix of hydrogenated fat, which recently displayed an estimated rumen bypass fraction of 86.6% after 24 h ruminal incubation (Watanabe et al., 2022). When supplementing lactating dairy cattle with this hydrogenated fat-embedded calcium gluconate (HFCG) product, we have observed increases in yields of milk fat (Seymour et al., 2021, 2023) and/or milk protein (Sanz-Fernandez et al., 2022; Seymour et al., 2022). Considering the low degree of rumen fermentation of HFCG, in addition to the similarities in responses to those achieved with postruminal infusion of calcium gluconate, we believe this supports our hypothesis that HFCG is able to escape the rumen in sufficient quantities to be fermented in the postruminal digestive tract in dairy cattle. Most of our work with HFCG to date has focused on cattle in mid-lactation in controlled research settings. Therefore, the objectives of this study were to evaluate the applications of HFCG in dairy cattle specifically during the transition period and early lactation under commercial conditions.

## Materials and Methods

All procedures used in this study were approved by the institutional animal care and use committee of Trouw Nutrition R&D, in compliance with European Directive 2010/63/EU. This experiment was executed at an 800-head commercial dairy farm in Durango, Mexico, between June and December 2019. All cattle were offered a common total mixed ration (Table 1) with fresh feed delivered at least 6× per day and had ad libitum access to water. The number of feedings per day was determined by farm management based on temperature and humidity index. Additionally, a single ration was offered to both dry and lactating cattle. The nutrient content of feeds was determined by near-infrared spectroscopy each month (Trouw Nutrition Mexico, Monterrey, Mexico). Cattle were milked 3× per day in a rotary parlor equipped to offer two choices of compound feed pellet based on animal ID, with an automated system in place to discard any pellet refusals from the manger upon the animal exiting the parlor. Individual yields of milk and milk components were measured at each milking using an inline milk analyzer (Afimilk, Afikim, Israel) and were used to calculate energy-corrected milk (**ECM**) yield as 0.01 × milk yield (kg/d) + 12.2 × fat yield (kg/d) + 7.7 × protein yield (kg/d) + 5.3 × lactose yield (Sjaunja et al., 1990). Bodyweight of cattle was recorded automatically when exiting the rotary parlor using an inline scale. Cattle were provided access to a sprinkler cooling system for approx. 45 min daily.

**Table 1.** Average ration composition and nutrient content. Values are presented on a % dry matter basis unless noted otherwise

Item	Mean content	Standard deviation
Ingredient^1^		
Rolled corn	30.7	0.30
Compound feed^2^	29.6	2.40
Corn silage	22.2	2.84
Alfalfa hay	16.6	3.82
Composition		
Dry matter, %	54.8	2.28
NDF	30.3	1.27
ADF	19.5	0.97
Crude protein	18.3	0.72
Starch	29.5	0.71
Fat	4.4	0.33
Ash	7.8	0.44
ME, Mcal/kg DM^3^	2.59	
NE_L_, Mcal/kg DM^3^	1.71	

^1^Oats were included in November at 1.33% DM; alfalfa silage was included in December at 4.76% DM.

^2^BFL Alta Producción 250 protein concentrate feed supplement (Agro Imperio SRL, Córdoba, Argentina).

^3^Calculated based on NASEM (2021) using average feedstuff nutrient composition and average ration formulation.

Data pertaining to the dates of first observed heat detection, first insemination, and confirmed pregnancy were also provided by the farm. Heat detection was performed both visually and with individual pedometers (AfiAct; Afimilk). Artificial insemination was performed by farm staff. Individual pregnancy checks were performed weekly by the herd veterinarian. When cattle were confirmed pregnant by the veterinarian, it was assumed that conception occurred on the date of the most recent insemination. Cattle were otherwise managed according to existing standard operating procedures at the farm, and any matters pertaining to cattle health and wellbeing, including vaccinations and medical interventions, were handled solely at the discretion of farm management and the herd veterinarian ensuring that there were no differences in management across treatment groups.

The experiment utilized a randomized complete block design with a 2 × 2 factorial arrangement of treatments. Multiparous dairy cattle with a body condition score of at most 3.5 (5-point scale) and at least 40 d before expected calving were enrolled in the study. Following enrolment, cattle were blocked (*n* = 4 per block) by parity (2 ± 1; mean ± SD), expected calving date, previous lactation 305 d milk yield (11.7 ± 2.05 tonnes), body condition score (3.4 ± 0.20) and body weight (702 ± 84.1 kg). Based on these parameters and assuming an average milk production of 25 kg/d at 50 DIM, we estimated the ME requirements for maintenance and lactation to be 20.6 Mcal/d and 24.7 Mcal/d, respectively (NASEM, 2021). After blocking, cattle entered a 7-d acclimation phase, starting at approximately 28 d before expected calving, during which they were offered 400 g of negative control (**CON; Table 2**) compound feed pellet once per day at approx. 1,100 h. During this acclimation phase, pellets were offered in the rotary parlor despite cattle not being milked. Individual feed refusals were measured during the last 4 d of the acclimation period, and any cattle that had cumulative refusals of over 50% were removed from the study. Following removal (four cattle total), blocks were readjusted prior to treatment randomization. A total of 164 animals entered the experimental phase of the study. It was confirmed that there were no statistically significant differences in blocking factors (*P* ≥ 0.054) between treatments at the start of the experimental phase.

**Table 2.** Formulation of negative control (CON) and hydrogenated fat-embedded calcium gluconate (HFCG) pellets. Values are expressed on a % as-is basis

Ingredient	CON	Prepartum HFCG	Postpartum HFCG
Soybean meal	85.9	81.8	84.4
Wheat bran	13.0	12.3	12.8
HFCG	0	4.7	1.60
Bentonite	1.0	0.96	0.99
Anise	0.10	0.10	0.10
Fast Green FCF (FD&C Green No. 3)^1^	0	0.10	0.10

^1^Food safe dye included to facilitate visual feed refusal scoring.

Treatments were randomly assigned to cattle within blocks and consisted of either hydrogenated fat-embedded calcium gluconate (**HFCG**) or CON compound feed pellet (Table 2) offered prepartum (**PRE**) and/or the first 100 d postpartum (**POST**), resulting in four treatment sequences: negative control both prepartum and postpartum (**CC**); HFCG both prepartum and postpartum (**GG**); HFCG only prepartum (**GC**); and HFCG only postpartum (**CG**). Beginning 21 d before expected calving, cattle were offered 400 g of either the CON or HFCG pellets once per day at approx. 1,100 h in the rotary parlor, despite not being milked. After calving, cattle were offered 400 g of either the CON or HFCG pellets at each milking for a total of 1,200 g/d. During both the PRE and POST periods, pellet refusals were visually assessed on randomly selected days by a technician blinded to treatments and scored on a scale of 1 to 4, where 1 indicated minimal to no refusals, 2 indicated up to 30% (120 g) remaining, 3 indicated between 30% and 50% (120 g to 200 g) remaining, and 4 indicated more than 50% (200 g or more) remaining. To evaluate differences in pellet refusals between treatments, the counts of each refusal score for each animal were calculated using the FREQ procedure of SAS v9.4 (SAS Institute Inc., Cary, NC). Refusal score was then analyzed by conditional logistic regression using the GLIMMIX procedure of SAS using maximum likelihood estimation with Laplace approximation. Additionally, sandwich estimators were requested using the EMPIRICAL = MBN option to reduce bias in the estimation of fixed effect standard errors (Kiernan, 2018). Counts of refusal scores were used as a weighting variable. The refusal score was modeled using a cumulative logit function assuming the data followed a multinomial distribution. The effects of prepartum supplementation, postpartum supplementation, and the interaction between the two were considered as fixed effects, while the effects of block and cow within a block were considered random. Negative covariance among blocks was accommodated using a compound symmetry covariance structure. Additionally, the length of the prepartum period (i.e., study enrolment to recorded calving date) was evaluated using a model considering the fixed and random effects as described above, assuming the data followed a Poisson distribution.

Fourteen cattle were removed from the study during the prepartum period for various health reasons: lameness (*n* = 2); severe dystocia (*n* = 2); abortion (*n* = 1); not stated (*n* = 9). Potential associations to prepartum treatment were evaluated using the GLIMMIX procedure of SAS, considering the effect of prepartum supplement (CON or HFCG) as fixed, and the effects of block and cow within block as random in a conditional model. Removal from the herd was treated as a binary distributed variable. After calving, an additional 42 animals were removed from the study for various reasons: lameness (*n* = 3); metritis (*n* = 3); left displaced abomasum (*n* = 2); postpartum complications (*n* = 2); abortion (*n* = 2); mastitis (*n* = 1); milk fever (*n* = 1); hardware disease (*n* = 1); cardiac arrest (*n* = 1); respiratory issues (*n* = 1); not stated (*n* = 25). The incidence of herd removal over the first 100 d of lactation was evaluated using two methods, where the days in milk at the last recorded milk yield were considered the day of removal from the herd. First, removal from the herd prior to 100 DIM was evaluated using the GLIMMIX procedure of SAS, considering the effects of prepartum supplement (CON or HFCG), postpartum supplement (CON or HFCG), and the interaction between the two as fixed effects, and the effects of block and cow within block as random effects in a conditional model. Herd removal was treated as a binary distributed variable. The same data were also analyzed using a shared frailty survival model with the PHREG procedure of SAS. Fixed effects were included as previously described, and the effect of the block was considered random.

To avoid potential bias in results due to cattle being removed from the study, only data from animals that remained in the study until at least 90 d of lactation (*n* = 108 total; CC = 28; CG = 27; GC = 21; GG = 32) were used for subsequent analyses. It was confirmed that there were no statistically significant differences in blocking factors associated with treatments following the removal of cattle from the study (*P* ≥ 0.117). Daily milk production data (e.g., milk and component yields) and body weight were averaged by week of lactation and analyzed using the GLIMMIX procedure of SAS. The effects of prepartum supplement (CON or HFCG), postpartum supplement (CON or HFCG), week of lactation and all interactions between them were considered as fixed, and the effects of block and cow within the block as random effects. A first-order autoregressive covariance structure was used to accommodate correlated errors due to repeated sampling on cattle across weeks of lactation. Fertility outcomes were analyzed using a similar model as described above, excluding the effect of week of lactation and associated interaction terms. The effect of cow within a block was modeled using a compound symmetry covariance structure to accommodate negative covariance among blocks, and it was assumed that all fertility outcomes followed a Poisson distribution.

For all analyses above, the Kenward–Roger correction was used to compute denominator degrees of freedom for tests of fixed effects, and a Newton–Raphson optimization with ridging was requested using the NLOPTIONS statement. The presence of potential interaction effects was evaluated by partitioning the main interaction effects into the underlying simple effects using the SLICE and SLICEDIFF options of the LSMEANS statement when the *P*-value for two-way interaction terms was ≤ 0.25 (Stroup et al., 2018); for models with a three-way interaction with time, main effects were first sliced by time and further analysis was conducted when multiple consecutive time points displayed evidence of potential PRE × POST interactions. Specific hypothesis tests were evaluated using the LSMESTIMATE statement. For all analyses, statistical significance was declared where *P* < 0.05, and trends where 0.05 ≤ *P* < 0.10.

## Results

The average length of the prepartum supplementation period was 26 ± 9.0 d (mean ± SD), with cattle assigned the CG treatment having a longer prepartum supplementation period (27 ± 1.7 d; mean ± SEM) relative to those assigned the CC treatment (23 ± 1.4; *P* = 0.045). It was confirmed there was no difference in refusals due to treatment (*P* ≥ 0.185; Table 3). No difference was observed in the culling rate prepartum between HFCG (11.0 ± 3.47%) and CON (6.1 ± 2.65%; *P* = 0.274) groups. There were no differences observed due to treatment for either odds of survival from calving to 100 DIM (*P* ≥ 0.274; Table 4) or the shared frailty survival analysis (*P* ≥ 0.233).

**Table 3.** Contingency table of supplement refusal scores in response to supplementation of hydrogenated fat-embedded calcium gluconate (HFCG) prepartum and/or postpartum, in multiparous Holstein dairy cattle. Values presented are counts of observed refusal scores

	PRE CON		PRE HFCG	
	POST CON	POST HFCG	POST CON	POST HFCG
Full study				
1: Minimal to no refusals	1035	1074	1003	1113
2: Up to 30% (120 g) remaining	491	476	407	515
3: 30% to 50% (120 g to 200 g) remaining	511	586	363	644
4: More than 50% (200 g +) remaining	833	937	616	843
Prepartum				
1: Minimal to no refusals	286	270	302	244
2: Up to 30% (120 g) remaining	106	122	88	115
3: 30% to 50% (120 g to 200 g) remaining	63	68	51	79
4: More than 50% (200 g +) remaining	53	61	31	45
Postpartum				
1: Minimal to no refusals	749	803	681	869
2: Up to 30% (120 g) remaining	385	354	316	400
3: 30% to 50% (120 g to 200 g) remaining	448	518	311	565
4: More than 50% (200 g +) remaining	769	876	583	796

^1^PRE CON: prepartum negative control; PRE HFCG: prepartum hydrogenated fat-embedded calcium gluconate; POST CON: postpartum negative control; POST HFCG: postpartum hydrogenated fat-embedded calcium gluconate.

**Table 4.** Survival from calving to 100 DIM (*n* = 150) and fertility outcomes (*n* = 108) in response to supplementation of hydrogenated fat-embedded calcium gluconate (HFCG) prepartum and/or postpartum, in multiparous Holstein dairy cattle. Values are presented as least squares means with standard errors of means in parentheses^1^

	PRE CON		PRE HFCG		*P*-values^2^		
	POST CON	POST HFCG	POST CON	POST HFCG	PRE	POST	PRE × POST
Survival to 100 DIM, %	65.8 (7.80)	61.6 (7.90)	55.9 (8.65)	69.2 (7.49)	0.912	0.573	0.274
Days to First Observed Heat, d	41.4 (3.91)	42.2 (4.05)	46.5 (5.04)	36.0 (3.20)	0.822	0.242	0.174
Days to First Service, d	76.8 (1.98)	79.3 (2.06)	74.1 (2.25)	75.0 (1.85)	0.097	0.409	0.708
Days to Confirmed Pregnancy, d	89.6 (8.84)	100.7 (12.28)	71.0 (11.44)	77.7 (10.19)	0.024	0.292	0.908

^1^PRE CON: prepartum negative control; PRE HFCG: prepartum hydrogenated fat-embedded calcium gluconate; POST CON: postpartum negative control; POST HFCG: postpartum hydrogenated fat-embedded calcium gluconate.

^2^PRE: effect of prepartum HFCG supplementation; POST: effect of postpartum HFCG supplementation; PRE × POST: prepartum-postpartum interaction effect.

Fertility outcomes are presented in Table 4. Days to first observed heat displayed evidence of a potential PRE × POST interaction, and it was confirmed that in cattle receiving HFCG prepartum, those that continued to receive HFCG postpartum (sequence GG) tended to go into heat earlier than those that switched to CON postpartum (sequence GC; *P* = 0.081). Additionally, cattle-supplemented HFCG prepartum tended to be serviced earlier (74.6 ± 1.46 vs. 78.0 ± 1.43 d, *P* = 0.097), and were confirmed pregnant earlier (74.3 ± 9.15 vs. 95.0 ± 8.40 d, *P* = 0.024) relative to the prepartum negative control.

Mean production outcomes are presented in Table 5, with full *P*-values including those for time-dependant interactions presented in Supplementary Appendix 1. It was determined that for the majority of outcomes (i.e., milk component contents, milk and lactose yields, and bodyweight), statistically significant differences were only observed at one to two individual time points for a subset of treatment sequences, and thus these interactions were deemed to be mainly due to the effect of time, rather than due to treatments. When considering the main treatment effects, no PRE × POST interactions were observed (Table 5).

**Table 5.** Lactation performance outcomes in response to supplementation of hydrogenated fat-embedded calcium gluconate (HFCG) prepartum and/or postpartum, in multiparous Holstein dairy cattle (*n* = 108). Values are presented as least squares means^1^

	PRE CON		PRE HFCG		SED^3^	*P*-values^2^		
	POST CON	POST HFCG	POST CON	POST HFCG		PRE	POST	PRE × POST
Milk yield, kg/d	27.7	28.7	27.6	28.3	1.21	0.780	0.291	0.831
Milk fat content, %	3.15	3.15	3.10	3.17	0.071	0.758	0.371	0.468
Milk lactose content, %	4.69	4.70	4.65	4.69	0.028	0.259	0.259	0.499
Milk protein content, %	3.14	3.19	3.11	3.17	0.057	0.492	0.125	0.923
Milk fat yield, g/d	851	896	840	881	34.7	0.576	0.068	0.928
Milk lactose yield, g/d	1303	1353	1292	1332	58.6	0.680	0.255	0.902
Milk protein yield, g/d	863	915	855	896	39.3	0.610	0.077	0.834
Energy-corrected milk yield, kg/d^4^	24.2	25.4	24.0	25.0	1.00	0.604	0.095	0.881
Bodyweight, kg	627	647	648	643	15.9	0.416	0.498	0.228

^1^PRE CON: prepartum negative control; PRE HFCG: prepartum hydrogenated fat-embedded calcium gluconate; POST CON: postpartum negative control; POST HFCG: postpartum hydrogenated fat-embedded calcium gluconate.

^2^PRE: effect of prepartum HFCG supplementation; POST: effect of postpartum HFCG supplementation; PRE × POST: prepartum–postpartum interaction. Interaction terms involving time were statistically nonsignificant save for yields of milk protein, milk fat, and energy-corrected milk. A complete list of *P*-values is provided in Supplementary Appendix 1.

^3^Standared error of differences.

^4^0.01 × milk yield (kg/d) + 12.2 × fat yield (kg/d) + 7.7 × protein yield (kg/d) + 5.3 × lactose yield (kg/d; Sjaunja et al., 1990).

**Figure 1.**
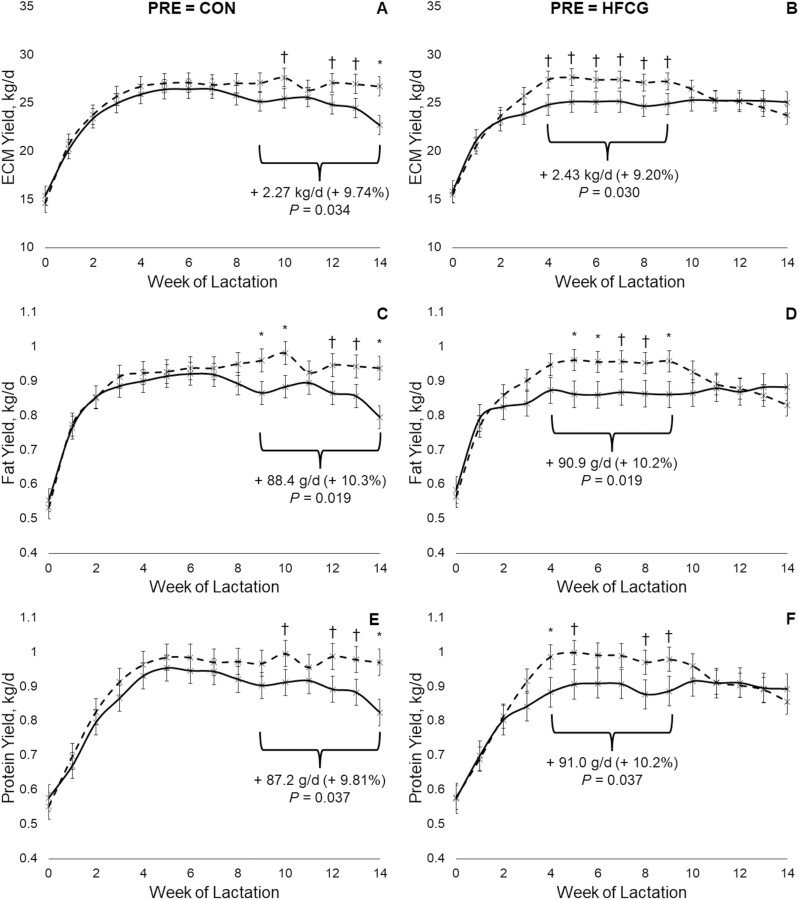
Average weekly yields of energy-corrected milk (ECM), fat and protein over the first 14 wks of lactation in multiparous Holstein cattle (*n* = 108) receiving either a negative control (CON) or hydrogenated fat-embedded calcium gluconate (HFCG) prepartum and/or postpartum. Panels on the left (A, C, and E) present animals assigned CON during the prepartum period. Panels on the right (B, D, and F) present animals assigned HFCG during the prepartum period. Solid lines indicate postpartum CON, dashed lines indicate postpartum HFCG, with error bars indicating standard errors of means. Brackets indicate the range pertaining to average response and *P*-value inset in the panel obtained using specific contrast. ^†^*P* < 0.10, ^*^P < 0.05. A complete list of *P*-values is provided in Supplementary Appendix 1.

Responses in yields of milk fat, protein, and ECM displayed evidence of PRE × POST × time interactions and are presented in Figure 1. Cattle that received CON prepartum displayed similar production levels from weeks 0 to 8 of lactation, after which those assigned the CG sequence had approx. 9% to 10% higher yields on average (*P* ≤ 0.037) relative to the CC sequence from weeks 9 to 14 (Figure 1, panels A, C, and E). Similarly, cattle that received HFCG prepartum displayed similar production levels from weeks 0 to 3 and 10 to 14 of lactation, while from weeks 4 to 9 those assigned the GG sequence had approx. 9% to 10% higher yields on average (*P* ≤ 0.037) relative to cattle assigned the GC sequence (Figure 1, panels B, D, and F). For cattle that received HFCG postpartum (sequences CG and GG), those assigned GG had lower or trends for lower yields (*P* ≤ 0.125) of milk fat, protein and ECM from weeks 12 to 14 of lactation relative to those assigned CG (Figure 1A). For cattle that received CON postpartum (sequences CC and GC), those assigned CG tended to have increased yields of milk fat and ECM (*P* ≤ 0.090) at week 14 only (Figure 1A and B).

## Discussion

Our group has previously observed increases in yields of milk fat (Seymour et al., 2021, 2023) and/or protein (Sanz-Fernandez et al., 2022; Seymour et al., 2022) to varying degrees in response to supplementing HFCG in lactating dairy cattle. In particular, Seymour et al. (2022) employed treatments synonymous with the GG and CC sequences used in the present study; while a time-dependant response to supplementation was also observed in multiparous cattle, increases in milk fat and protein yield occurred over the first 8 wks of lactation. We have confirmed that while no differences between the CC and GG sequences for any outcome over the first 8 wks of lactation were found (*P* ≥ 0.274), the magnitude of the responses in yields of milk and milk components observed, while observed later in lactation, were consistent with those reported by Seymour et al. (2022; approx. 8% increase). Additionally, the responses in milk fat and protein yield were higher than those in previous studies (Seymour et al., 2021, 2023; Sanz-Fernandez et al., 2022); this could be due in part to the lower baseline production level in the current study (approx. 25 kg/d in established lactation) relative to previous studies (~30 to 40 kg/d).

To our knowledge, this is the first study to evaluate reproductive performance in response to supplementation of calcium gluconate or other salts of gluconic acid. It is generally accepted that indicators of negative energy balance (**NEB**) are associated with decreased fertility (Cardoso et al., 2020). The results relating to days to first service and days to confirmed pregnancy following prepartum supplementation of HFCG are noteworthy. In particular, the similarities between the estimated days to first service and days to confirmed pregnancy suggest that HFCG improved the first service conception rate. The meta-analysis of Civiero et al. (2021) suggested that for every 10 MJ ME increase in average daily NEB over the first 3 weeks of lactation, the first observed heat was delayed by 0.8 d in multiparous cattle. This could indicate that animals assigned the GG treatment sequence had improved energy status in early lactation relative to those assigned the GC sequence. As discussed below, this could be associated with the mediation of a localized inflammatory response in the gastrointestinal tract, or other aspects that could improve energy balance and thus fertility.

The precise mode of action of HFCG in the hindgut remains unclear. The main hypothesis is that the active ingredient in HFCG (gluconate) acts as a precursor for acetate and/or butyrate in the hindgut (Asano et al., 1994; Tsukahara et al., 2002, 2006). Some groups have investigated the potential impacts of gluconate supplementation on gut health & morphological measures with mixed results (Poeikhampha and Bunchasak, 2011; Michiels et al., 2020; Watanabe et al., 2022), while others have proposed that luminal VFAs may act as signaling molecules to affect extragastrointestinal responses (Stilling et al., 2016; O’Riordan et al., 2022), such as energy partitioning. In previous studies with HFCG, we have proposed that responses in milk protein yield were likely supported by improved voluntary dry matter intake (Seymour et al., 2022), while responses in milk fat yield were most likely supported by increased uptake of preformed fatty acids of endogenous origin by the mammary gland (Seymour et al., 2021). As feed intake nor blood per milk metabolites were measured in the present study, we can only speculate as to the underlying nature of the observed responses. However, the findings of this study and others with the same (e.g., Seymour et al., 2022, 2023) or similar products/compounds (Doelman et al., 2019; McKnight et al., 2019; Seymour et al., 2021) demonstrate the beneficial effects of HFCG supplementation and may provide insights on how suboptimal gastrointestinal health can affect overall animal performance and energy partitioning.

In conclusion, prepartum supplementation of HFCG reduced the time to confirmed pregnancy in lactating multiparous Holstein dairy cattle. Cattle that continued HFCG supplementation for the first 100 DIM demonstrated increased yields of milk fat, protein and ECM from weeks 4 to 9 of lactation relative to those that stopped HFCG supplementation after calving. Cattle that received HFCG solely during the first 100 DIM demonstrated improved yields of milk fat, protein and ECM from weeks 9 to 14 of lactation. Based on these findings, the inclusion of HFCG both in close-up and early lactation rations may improve production and fertility in lactating multiparous dairy cattle.

## Supplementary data

Supplementary data are available at *Translational Animal Science* online.

txad104_suppl_Supplementary_Appendix_S1
