# Supplementary material for: Evaluating lactation performance of multiparous dairy cattle to prepartum and/or postpartum supplementation of fat-embedded calcium gluconate
Source: Transl Anim Sci. 2023 Aug 31;7(1):txad104. doi: 10.1093/tas/txad104 (PMC10494878; doi:10.1093/tas/txad104)
Supplement: txad104_suppl_Supplementary_Appendix_S1 [file txad104_suppl_supplementary_appendix_s1.docx]

Appendix 1: Complete p-values for treatment-by-time interactions<?Double?>

| <?Char=Text?> | PRE<?Char=Decimal?> | POST<?Char=Decimal?> | PRE ×  × POST<?Char=Decimal?> | PRE ×  × WOL<?Char=Decimal?> | POST ×  × WOL<?Char=Decimal?> | PRE ×  × POST ×  × WOL<?Char=Decimal?> |
| --- | --- | --- | --- | --- | --- | --- |
| Milk yield, kg/d | 0.780 | 0.291 | 0.831 | 0.998 | 0.419 | 0.165 |
| Milk fat content, % | 0.758 | 0.371 | 0.468 | 0.938 | 0.554 | 0.132 |
| Milk lactose content, % | 0.259 | 0.259 | 0.499 | 0.326 | 0.771 | 0.199 |
| Milk protein content, % | 0.492 | 0.125 | 0.923 | 0.927 | 0.858 | 0.435 |
| Milk fat yield, kg/d | 0.576 | 0.068 | 0.928 | 0.891 | 0.295 | 0.182 |
| Milk lactose yield, kg/d | 0.680 | 0.255 | 0.902 | 0.999 | 0.732 | 0.156 |
| Milk protein yield, kg/d | 0.610 | 0.077 | 0.834 | 0.999 | 0.689 | 0.310 |
| Energy-corrected milk yield, kg/d | 0.604 | 0.095 | 0.881 | 0.990 | 0.566 | 0.222 |
| Bodyweight, kg | 0.416 | 0.498 | 0.228 | 0.169 | 0.896 | 0.169 |

1PRE: effect of prepartum supplementation; POST: effect of postpartum supplementation; PRE ×  × POST: prepartum ×  × postpartum interaction; PRE ×  × WOL: prepartum × × week of lactation interaction; POST ×  × WOL: postpartum ×  × week of lactation interaction; PRE ×  × POST ×  × WOL: prepartum ×  × postpartum ×  × week of lactation interaction.
